# Hydrogen Production from Energy Poplar Preceded by MEA Pre-Treatment and Enzymatic Hydrolysis

**DOI:** 10.3390/molecules23113029

**Published:** 2018-11-20

**Authors:** Karolina Kucharska, Rafał Łukajtis, Edyta Słupek, Hubert Cieśliński, Piotr Rybarczyk, Marian Kamiński

**Affiliations:** 1 Department of Process Engineering and Chemical Technology, Faculty of Chemistry, Gdańsk University of Technology, Narutowicza 11/12 Street, 80-233 Gdańsk, Poland; rafal.lukajtis@pg.edu.pl (R.Ł.); edyta.slupek@pg.edu.pl (E.S.); piotr.rybarczyk@pg.edu.pl (P.R.); marian.kaminski@pg.edu.pl (M.K.); 2 Department of Molecular Biotechnology and Microbiology, Faculty of Chemistry, Gdańsk University of Technology, Narutowicza 11/12 Street, 80-233 Gdańsk, Poland; hcieslin@pg.edu.pl

**Keywords:** hydrogen, dark fermentation, Box-Behnken design, enzymatic hydrolysis, MEA pre-treatment

## Abstract

The need to pre-treat lignocellulosic biomass prior to dark fermentation results primarily from the composition of lignocellulose because lignin hinders the processing of hard wood towards useful products. Hence, in this work a two-step approach for the pre-treatment of energy poplar, including alkaline pre-treatment and enzymatic saccharification followed by fermentation has been studied. Monoethanolamine (MEA) was used as the alkaline catalyst and diatomite immobilized bed enzymes were used during saccharification. The response surface methodology (RSM) method was used to determine the optimal alkaline pre-treatment conditions resulting in the highest values of both total released sugars (TRS) yield and degree of lignin removal. Three variable parameters (temperature, MEA concentration, time) were selected to optimize the alkaline pre-treatment conditions. The research was carried out using the Box-Behnken design. Additionally, the possibility of the re-use of both alkaline as well as enzymatic reagents was investigated. Obtained hydrolysates were subjected to dark fermentation in batch reactors performed by *Enterobacter aerogenes ATCC 13048* with a final result of 22.99 mL H_2_/g energy poplar (0.6 mol H_2_/mol TRS).

## 1. Introduction

Hydrogen is regarded as a fuel of the future due to its vast abundance and the possibility of its sustainable production. However, hydrogen does not occur in Nature in its elemental form and thus hydrogen production requires the use of various conversion technologies. Methods of hydrogen production include fossil fuel reforming, coal gasification, plasma arc decomposition of fossil fuels, water electrolysis, photocatalysis, photo-electrolysis or dark fermentation [1]. Among the above-mentioned methods, dark fermentation is believed to be the most promising method of hydrogen production from renewable energy sources as the net energy ratio for this method is equal to 1.9 [2,3].

Production of hydrogen via dark fermentation from various biomass feedstocks is widely reported in the literature [4,5,6,7,8,9,10]. Dark fermentation enables a low energy input production of hydrogen from renewable feedstocks [11]. Various lignocellulosic materials may serve as substrates for hydrogen production, including cornstalk, rice straw, wheat straw, grass, switchgrass, sugarcane bagasse, *Miscantus*, energy willow, and energy poplar leaves [12,13,14,15,16,17,18]. However, efficient fermentative hydrogen production from the abovementioned substrates requires their pre-treatment and efficient saccharification.

The need for lignocellulosic biomass pre-treatment prior to dark fermentation results primarily from the composition of lignocellulose. The complex chemical structure of the raw materials (Table 1) necessitates the initial pre-treatment step, usually by means of chemical hydrolysis. The purpose of the pre-treatment is to destroy the structure of cellulosic biomass plant cell walls and to make cellulose and hemicellulose more accessible to the subsequent enzymatic hydrolysis process. In general, the plant cell wall is a heterogeneous mix of polymers that constitutes a matrix in which above mentioned carbohydrates are encapsulated. The performance of enzymatic hydrolysis of cellulose and hemicellulose in the cell wall is highly dependent on the pre-treatment operations. In this perspective, one of the most effective pre-treatment method is the alkaline pre-treatment which enhances the rate of enzymatic hydrolysis and the yield of TRS as fermentable sugars, including mainly glucose and xylose, released to the hydrolysate [10]. Pre-treatment of lignocelllulosic biomass may be realized by means of various methods including physical, chemical, physicochemical and other methods, depending on the type of substrate and the resulting final products [19,20,21].

Efficient fermentation of biomass must be preceded by at least two steps, i.e., biomass pre-treatment and saccharification. Saccharification may be realized e.g., either by acid or enzymatic hydrolysis [20]. Enzymatic hydrolysis is carried out by highly specific cellulase enzymes to yield reducing sugars, including glucose [23]. The rate of enzymatic hydrolysis of lignocellulosic residues is limited by many factors such as the degree of polymerization of the raw material, the moisture content, hemicellulose and lignin content or porosity of the raw material [24,25]. Thus, the efficiency of the enzymatic hydrolysis is primarily dependent on the results of pre-treatment operations. The main goals of pre-treatment are: (i) to achieve a high degradation of sugars (including those derived from hemicellulose), (ii) to minimize the formation of inhibitors for subsequent fermentation step, (iii) to remove lignin derivatives and possibly recover value-added products from hydrolysates, (iv) to reduce energy consumption [19,26]. Pre-treatment of raw materials should also provide a reduction of the cellulose crystallinity [27] and consequently an increase of the contact surface between raw material and enzymes [28,29]. Various approaches towards pre-treatment of lignocellulosic materials is presented in Table 2.

Studies on the direct conversion of lignocellulosic materials to H_2_ exist, however most microorganisms require pre-treated biomass residues as substrates for dark fermentation [30,31,32,33,34,35,36]. The degree of pretreatment depends on the nature of the raw material and on the type of inoculated microorganism [37]. Pre-treatment has great improvement potential when the monosugar-releasing efficiency from cellulose and hemicellulose is considered. However, the pre-treatment also strongly influences the total process cost by reducing enzyme loading, mixing power, waste treatment demands and power generation [38]. The conversion of cellulose and hemicellulose is catalysed by cellulases and hemicellulases, respectively. Cellulases are usually a mixture of several enzymes. The three predominant ones are: endo-1,4-β-glucanase, which hydrolyzes the inner β-1,4-glycosidic bonds; exo-1,4-β-glucanase, which removes glucose or cellobiose from the free chain-ends; and β-glucosidase (cellobiase), which hydrolyzes cellobiose [25,39,40,41]. Cellulose hydrolysis starts with adsorption of cellulase enzymes onto the surface of cellulose. Afterwards, cellulose is biodegraded to the fermentable sugars and cellulase is desorbed from the biomass surface.

Cellulose microfibers are surrounded by hemicellulose polysaccharides. Therefore, auxiliary enzymes that attack hemicellulose are also used in the saccharification process. In the first step, endo-1,4-β-xylanase depolymerizes xylan into xylooligosaccharides. Further, xylanases, such as β-glucuronidase, α-arabinofuranosidase, and acetyl xylan esterase, cleave side chains and side groups of heteroxylan, while galactomannanase and glucomannanase hydrolyze glucomannan [12,47]. The optimal balanced combination of enzymes is needed to effectively modify the complex structure of lignocellulosic materials.

The main advantages of enzymatic vs. acid hydrolysis of lignocellulosic biomass are the elimination of corrosion problems and mitigation of fermentation inhibitor formation, such as hydroxymethylfurfural (HMF), furan, furfural, levulinic acid and formic acid [48,49]. The drawbacks of enzymatic hydrolysis include high cost of enzymes, relatively long reaction times and the necessity to separate the hydrolysis products from the enzymes [50]. Enzyme immobilization and recycling are solutions to both the mentioned problems [51]. Enzyme immobilization enables the easy separation of the enzymes after the hydrolysis as well as their possible reuse. The recycling of Eupergit C-immobilized β-glucosidase revealed that the stability of enzymes was maintained during five successive rounds of enzymatic hydrolysis [50,51]. Among a number of possible carriers used for enzyme immobilization, after examining the present state of art, we decided to use diatomaceous earth (DE) for this purpose. The choice of DE as a carrier was determined by its following properties. It is a material of natural origin, relatively cheap and with a high content of silica. What’s more, it has a high porosity, developed surface, low density, low conduction coefficient and is chemically inert. Moreover, the enzyme can be immobilized on the DE surface either by adsorption or by covalent binding to the functional groups present on the DE surface. What was particularly interesting to our research team was the possibility of modifying the structure of DE leading to give them magnetic properties. This allows for effective removal of the immobilized enzyme from the bioreactor after its use with the use of magnetic biosorption technology [52,53]. In our opinion, in future studies, the magnetic DE nanoparticles technology gives an interesting opportunity for developing novel chemically modified DE carriers for enzymes immobilization.

Each step of biomass treatment affects its consecutive processing. The harsh pH conditions of chemical hydrolysis, for example, require further pH adjustment before the enzymatic hydrolysis and may also result in the formation of undesired products [20,54,55,56,57]. Thus, the harsh conditions of acid pre-treatment may lead to the degradation of monomeric sugars into by-products such as 5-hydroxymethylfurfural or furfural, which may inhibit the fermentative hydrogen production [10]. On the other hand, alkaline pre-treatment is mostly effective for low lignin content biomass [19]. It is therefore reasonable to investigate other chemicals that may be of use for efficient biomass alkaline pre-treatment. Monoethanolamine (MEA), is an interesting example of a reagent for such a purpose. It maintains mild pH conditions during chemical hydrolysis and it is cost-effective if compared to hydrolysis with ammonia. MEA has been applied for the removal of lignin from woody biomass as a component of an ionic liquid [58]. 

The aim of the present work is threefold: (i) to investigate the effectiveness and optimize the conditions of chemical hydrolysis of the terrestrial part of energetic poplar (EP) with MEA, (ii) to investigate the possibility of reusing MEA solution for further chemical hydrolysis, (iii) to investigate the possibility and resulting efficiency of recycling of the enzymes. The selection of the substrate (branches and trunk elements of energetic poplar) is justified by its popularity towards biofuels production, low soil and water requirements as well as relatively fast growth [18,59,60].

## 2. Materials and Methods

### 2.1. Biomass Characterization

The energy poplar (EP) used in this study was obtained from a local producer (Wejherowo, Poland). The terrestrial part of the plant was used. The composition of EP was determined according to NREL procedures [61,62,63,64,65].

Milled and minced biomass (garden shredder 425, Meec Tools, 729-162, Jula AB, Skara, Sweden and an Ultra Centrifugal Mill ZM 200 EP, RETCH, Verder Scientific, Dusseldorf, Germany) was sieved through a 0.75 mm screen. The material after the grinding was dried and stored at room temperature in sealed containers. Prior to the alkaline pre-treatment, the material was dried in a laboratory dryer at 105 °C for 4 h and then stored in a desiccator with NaOH flakes (drying agent).

### 2.2. Analytical Methods

Total solids, ash and extractives were determined according to the National Renewable Energy Laboratory (NREL) analytical procedures [61,62,63,64,65]. The content of cellulose and hemicellulose was determined by HPLC with a Rezex Pb^2+^ column (Phenomenex, California, CA, USA, 300 × 7.8 mm, 8 μm) and a refractometric detection (Knauer Smartline RID 2300, Berlin, Germany,). Water with a flow rate of 0.6 mL/min was used as the eluent. The EP composition was found to be 39.5% cellulose, 22.2% hemicellulose, 26.3% lignin, 0.1% ash, 3.5% water, 8.4% ethanol extractives, 6.4% water extractives.

The presence and the concentration of reducing monosugars and disugars (glucose, xylose, arabinose, mannose, galactose and cellobiose) were determined using HPLC with a Rezex Pb^2+^ column and a refractometric detector (1755 Refractive Index Monitor, Bio-Rad Model 1755). Mobile phase was water, flow: 0.6 mL/min, column temperature was set at t = 80 °C.

The volatile fatty acids and alcohols in the fermentation broth and fermentation inhibitors (furfural, 5-HMF, and levulinic acid) in the hydrolysates were determined using HPLC with a Shodex SH1011 (city, Japan) column and a refractometric detection (Knauer). Mobile phase was 5 mM H_2_SO_4_ solution, 0.6 mL/min flow, column temperature was set at t = 60 °C. Gaseous products of fermentation (H_2_ and CO_2_) were analyzed using a gas chromatograph (AutoSystem XL, Perkin-Elmer, Norwalk, CT, USA) equipped with a Porapak Q column (Sigma-Aldrich, Merck, Darmstadt, Germany), 100–120 mesh, 6.5 m × 1/8) and a thermal conductivity detector (TCD). Oven temperature was set at 60 °C. Turbochrom software was used for recording and processing of chromatograms. Gas samples were taken from the reactor during the lag, exponential and decline phases of culture growth.

The pH was continuously monitored during the fermentation (Arduino Microcontroller data-logger, Continuous pH regulative system developed at Gdańsk University of Technology, Gdańsk, Poland). The growth of *E. aerogenes* ATCC 13048 culture was monitored by measuring of OD^−^ at λ = 600 nm (optical density of culture). Concentration of total phenolic compounds was determined by UV-VIS. The calibration curve was prepared for vanillin [66,67,68,69,70]. The absorbance measurement was made at λ = 280 nm. To obtain the sum of the phenolic compounds, the concentrations of furfural and 5-HMF (determined by HPLC) were subtracted from the concentrations determined by the UV-VIS method [66,67,68,69,70].

### 2.3. Alkaline Pre-Treatment 

Alkaline pre-treatment was carried with MEA solution. In this work, the influence of three variable process parameters (reaction time, MEA concentration, temperature) on the yield of reducing sugars obtained as a result of lignocellulosic biomass saccharification was examined by means of RSM (Table 3). 

During the alkaline pre-treatment, 20 mL of catalyst solution was used per each gram of lignocellulosic material. The reactions were carried out in 100 mL glass reactors. Details are listed in Table 3. After the pre-treatment, the slurry was filtered through Buchner funnel to separate the solid fraction. The solid residue was washed three times by water and two times by acetone. In addition, the alkaline pre-treatment experiment was carried out with repeated use of the catalyst solution (threefold).

**Figure 1:**
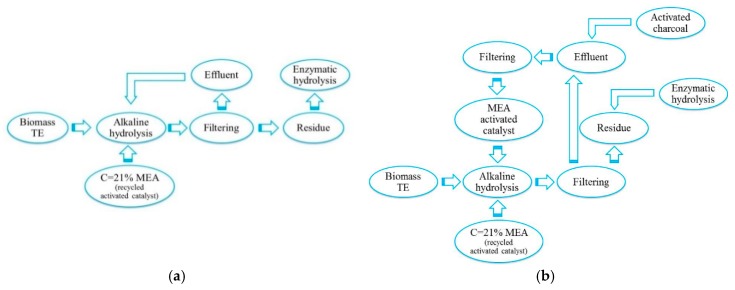
Schematic diagram of MEA catalyst re-use (**a**). direct approach (**b**). purifying approach.

To this end, experiments were carried out using two approaches. The first consisted in the direct reuse of the catalyst solution for the pre-treatment of the new lignocellulosic feed (Figure 1a.) while the second approach included an additional step of purifying the alkaline solution prior to reuse for further processing (Figure 1b). For this purpose, activated carbon was used to remove chemical compounds from the catalyst solution.

### 2.4. Enzymatic Hydrolysis

After completion of the pre-treatment with MEA, the pre-treated EP samples were further processed using biochemical methods i.e., by enzymatic hydrolysis. In this study, high quality enzyme preparations for the hydrolysis of lignocellulosic residues were used. Enzyme immobilization on diatomite was applied, taking into account both the need for the removal of enzymes prior to fermentation and the possibility of immobilized enzyme separation by centrifugation [55]. 

According to the manufacturer′s declaration, the supplementation of cellulolytic enzyme mixtures with an additional portion of β-glucosidase may increase the hydrolysis yield to monosaccharides, due to the fact, that cellobiose is an inhibitor of cellulolytic enzymes [71]. Therefore, in this commercially available cellulolytic enzyme mixtures Viscozyme L (Novozymes Corp., Copenhagen, Denmark) supplemented with commercially available glucosidase (Sigma-Aldrich, Stockholm, Sweden) were used. The immobilized enzyme preparation consisted in placing 20 mL of enzymatic solution (Viscozyme L: glucosidase from *Aspergillus niger*; 0.95:0.15 *m*/*m*) in a 50 mL beaker with 2.5 g dry diatomite and stirring the solution for 1 h at low speed on a magnetic stirrer at room temperature. The diatomite with the immobilized enzyme was washed in a column with a small amount (about 10 mL) of McIlvaine’s buffer. The bed stabilized after 20 min. Next, the diluted diatomite was stored under a layer of buffer (about 5 mm).

Milled and minced EP after alkaline pre-treatment (0.2 g) was added to the flasks and supplemented with a suspension of cellulolytic enzymes immobilized on diatomite to 10 mL. The reaction flasks were incubated in a shaker at 42 °C for 24 h. Subsequently, samples were taken, the enzyme containing bed was separated by centrifugation and filtration, and the contents of monosaccharides and cellobiose in the supernatant solution were analyzed. Control experiments were carried without the addition of EP. For the use of the enzymatic hydrolyzing preparations, the immobilized bed was shaken and then introduced to the solid residue of lignocellulosic biomass solution after alkaline pre-treatment. To recover the enzymes after enzymatic hydrolysis, diatomite was separated from EP residues and double washed using McIlvaine’s buffer, and then dried for 24 h in 42 °C.

### 2.5. Design of Experiments 

The response surface methodology (RSM) was used to determine the optimal alkaline pre-treatment conditions resulting in the highest total released sugar yield. Three variable parameters (temperature, MEA concentration, time) were selected to optimize the alkaline pre-treatment conditions. The research was carried out using the Box-Behnken design. The plan includes 15 experiments with different levels of three variables (Table 2) used to determine the influence of each of the variable parameters and their mutual interactions on the TRS obtained from one gram of biomass. While using the Box-Behnken design, a high concentration of points in the optimal area is obtained. This allows for a correct selection of the most favorable process parameters. The authors wanted to avoid the situation where the corner points in the central composite design are very extreme, i.e., they are at the highest level of several factors. The R-studio [72] software was used to determine and evaluate the coefficients of the regression model equation and their statistical significance. Box-Behnken designs are efficient designs for fitting second polynomials to response surfaces, because they use relatively small number of observations to estimate the parameters. Rotatability is a reasonable basis for the selection of a response surface design. The purpose is the optimisation when the location of the optimum is unkown, therefore it makes sense to use a design that provides equal precision of estimation in all directions. Experimental data was evaluated using three-dimensional diagrams showing the response surface area. RSM results are presented using MS Excel. In order to match the dependences of the parameter variables with the response value, the second order polynomial Equation (1) presented in the general form was applied:

(1) $$Y_{TRS}=\beta_{0}+\sum \beta_{i}x_{i}+\sum \beta_{\mathrm{ij}}x_{i}x_{j}+\sum \beta_{\mathrm{ii}}x_{i}^{2}$$

Y_TRS_ (mg/g_biomass_) is the expected value of TRS after two-stage alkaline pre-treatment and enzymatic hydrolysis, X_i_ (X_1_, X_2_, X_3_) describe values of independent variables of the alkaline pre-treatment process, catalyst concentration, temperature and time, respectively β_0_ is the intercept value, β_i_ (β_1_, β_2_, β_3_) stand for linear coefficients, β_ij_ (β_12_, β_23_, β_13_) for coefficients of mutual interaction of parameters, β_ii_ (β_11_, β_22_, β_33_) for squared linear coefficients. 

### 2.6. Dark Fermentation

**Figure 2:**
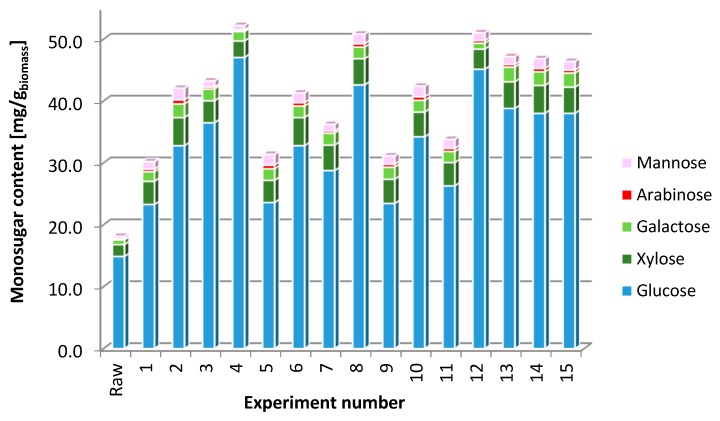
Content of monosugars in raw EP and alkaline pre-treated enzymatic hydrolysates corresponding to the conditions of experiments presented in Table 4.

*Enterobacter aerogenes* ATCC 13048 (Selectrol TCS Biosciences Ltd., Buckingham, UK) was used as a model microorganism for hydrogen production in dark fermentation process. The dark fermentation was carried out in triplicate in sterile 1200 mL glass bioreactors with an initial working volume of 1 L fermentation broth and under regulated pH conditions. The initial fermentation broth was composed of an appropriate portion of a sterile minimal medium Buffered Peptone Water (Biomaxima, Gdańsk, Poland) and EP hydrolysate. The total sugar concentration (see Table 4), utilized as a sole carbon source by *Enterobacter aerogenes* ATCC 13048, in the initial fermentation broth was set as 5.5 g/L. The proportions of sugars in hydrolysates obtained during optimization are presented in the Figure 2. For the purposes of fermentation, an optimal hydrolysate *H_opt_* was used. The sugar content in *H_opt_* is equal to 528.7 mg/g_biomass_ (including 462 mg/g_biomass_ glucose, 26,15 mg/g_biomass_ xylose, 14.97 mg/g_biomass_ galactose, 2,4 arabinose, 7,19 mg/g_biomass_ cellobiose). The initial pH of fermentation broth was adjusted to 7.00 with 1 M NaOH.

Next, each bioreactor was inoculated with 100 mL of pure bacterial cultures of *Enterobacter aerogenes* ATCC 13048 (inoculum OD_600_ = 2435) propagated in sterile Thioglycollate Broth Alternative (Biomaxima, Gdańsk, Poland) at 37 °C with an agitation (280 RPM). Before inoculation, anaerobic conditions for *Enterobacter aerogenes* growth in bioreactor were created by purging the reactors with sterile nitrogen gas for 60 min. Operational set-points were set at 37 °C and 320 RPM for temperature and agitation, respectively. The pH control system in bioreactor was connected with a peristaltic dosage pump dispensing a portion of 1 M NaOH when the pH drifted below the set-point value (7.00 ± 0.1). Experiments were carried for a process time of 72 h which corresponds to the late logarithmic death phase of *Enterobacter aerogenes* culture.

## 3. Results and Discussion 

### 3.1. Effect of Alkaline Pre-Treatment on the Biomass Content 

In order to determine the effects of the MEA pre-treatment on the change of biomass composition, the weight loss (biomass recovery), as well as the cellulose, hemicellulose and lignin content, lignin removal and cellulose and hemicellulose recovery were determined in the residues after the treatment (Table 4). The information regarding conditions for each run of the experiment correspond with the ones presented in Table 5. The range of variables is given in Table 3.

The results of investigations indicate that it is not possible to completely remove lignin from the plant material as a result of MEA pre-treatment, even if high temperatures and high concentrations of MEA are used. Application of optimal conditions (exp. 4, Table 4) favors delignification because the lignin contractiveness is reduced from 26.3% in the raw material to 15.7%. Similar results can be found in the literature [73]. A decrease in lignin content from 31.7% to 12.07% due to the treatment using microwaves and 0.4 M NaOH solution at 170 °C for 7 min was obtained [73]. The inability to completely remove lignin from the processed material may contribute to a reduced efficiency of the enzymatic hydrolysis. The highest weight loss (30.5%) was observed for the process carried out at 100 °C and for MEA concentration of 11% (*w*/*v*) for 11 h. The weight loss corresponds to the high removal rates of lignin (60.2%) and hemicellulose (52.2%). It was observed that for mild conditions, as the MEA concentration is considered, the loss of hemicellulose [74] and lignin is insignificant, and the change in the chemical composition of the material is probably mainly due to dissolution of the extractives in the solution.

### 3.2. Effect of Alkaline Pre-Treatment on the Saccharification Efficiency

Among the known methods for the pre-treatment of lignocellulosic materials, alkaline pre-treatment is one of the most effective methods for increasing the concentration of reducing sugars in the hydrolysates [38,59]. In this work, monoethanolamine (MEA) was used for alkaline pre-treatment. This compound is a promising processing catalyst; the effectiveness of its use on the performance of reducing sugars has also been investigated. The concentration of reducing sugars, especially glucose, after enzymatic hydrolysis was used as a reference. The TRS in the hydrolysates after alkaline pre-treatment and enzymatic hydrolysis was up to 5.84 times higher when compared to the hydrolysates obtained during one-step enzymatic hydrolysis of untreated raw material. The best digestibility of cellulose (92.0%, exp. 4, Table 5) was achieved for the pre-treatment at 100 °C, pre-treatment time of 11 h and MEA concentration of 25% (*v*/*v*). For these conditions, the highest loss of lignin and biomass were also observed (Table 4). Usually, higher cellulose digestibility is related to the degree of lignin [25,26] and hemicellulose [27,28] removal. However, a relatively high glucose yield (71%) can already be achieved at a lower process temperature (exp. 2, Table 5). It is possible to achieve the performance close to maximal for both the lower temperature (exp. 8, Table 5.) and the catalyst concentration (exp. 12, Table 5.), but it is necessary to extend the process time from 11 to 20 h.

The efficiency of the saccharification often depends on the degree of lignin removal [29]. It turns out that higher glucose concentration in the hydrolysate corresponds with a higher degree of lignin removal. The conditions of the applied pre-treatment have a significant impact on the mass of the solid residue after the pre-treatment [75,76]. The remaining mass of the EP after delignification oscillated between 69.5% and 88.1% in relation to raw materials mass. Biomass yield after pre-treatment decreases with both temperature and catalyst concentration increase. 

The maximum sugar yield increased in relation to that obtained from the untreated biomass by 2.7 times under the optimal conditions. Literature [56,77] provides information about up to 6.3 times productivity increase, but this is related to other processing conditions, i.e., 2% NaOH, 30 min, 121 °C, and the other lignocellulosic material (wheat straw). In other studies, the use of RSM in the optimization of the treatment conditions using ammonia solution as a catalyst and giant red as a substrate allowed to obtain TRS yield 538.1 g/g dry biomass for process conditions T = 170 °C, ammonia to biomass ratio 538.1 g/kg, water to biomass ratio 0.8 g/g and time 10 min [14].

A detailed content of monosugars in the TRS is presented in the Figure 2. For the efficiency comparison, the composition of the hydrolysate obtained for raw EP after enzymatic saccharification was presented (Raw). 

### 3.3. Effects of Variables on the Glucose Yield

The values of TRS ranged from 298.2 to 515.5 mg/g_biomass_ depending on the reaction conditions. The obtained results, compared to the concentration of sugars (181.4 mg/g_biomass_) obtained from direct enzymatic hydrolysis of raw material, indicate a significant influence of the applied conditions for the treatment of lignocellulosic material. According to data presented in Table 4, a quadratic model was proposed with the view of further presentation of response surface area. Evaluation of the models is based on the F-test. When the *p*-value for the model was less than 0.01, it indicated that the model was statistically significant. The obtained results (Table 5) and the analysis of the independent variables allowed to obtain a model described by the equation (Equation (2)). The values of the equation coefficients and their statistical assessment are presented in Table 6. The *p*-value for the model is equal to 1.02 × 10^−5^, and was lower than 0.001, which in combination with the F value of 44.5, indicates that the obtained model is statistically significant. In the square equation only significant variables of value are included:

Y_TRS_ = −25.654 + 12.322X_1_ + 5.522X_2_ + 12.074X_3_ − 0.224X_12_− 0.029X_22_ − 0.562X_32_ + 0.054X_2_X_3_ (2)

*p*-value less than 0.05 made it necessary to reject the X_1_X_2_ and X_1_X_3_ interactions in the next iterative steps. The determination coefficient (R^2^) was 0.949. 

**Figure 3:**
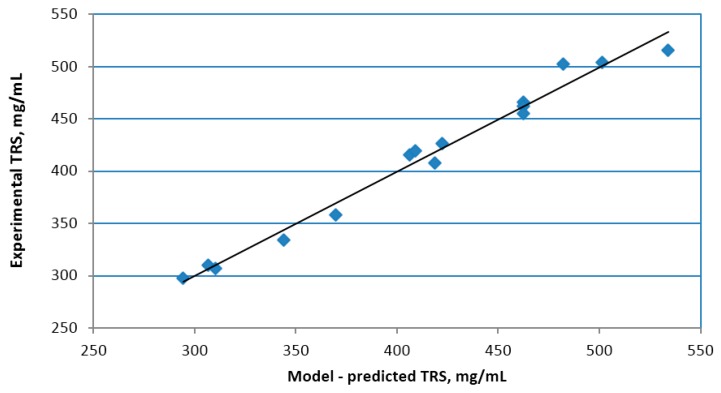
Analysis of variance for the theoretical (model-predicted) and experimental TRS yield.

The quality of the model has been additionally verified by comparing the predicted values with the results obtained, the relation is shown in Figure 3. The determination coefficient is R^2^ = 0.982.

### 3.4. Optimization of the Pre-Treatment Conditions

**Figure 4:**
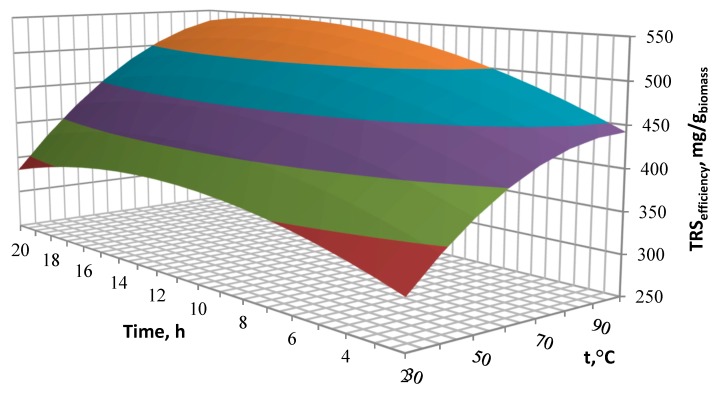
Influence of the pre-treatment time and temperature on the TRS efficiency.

**Figure 5:**
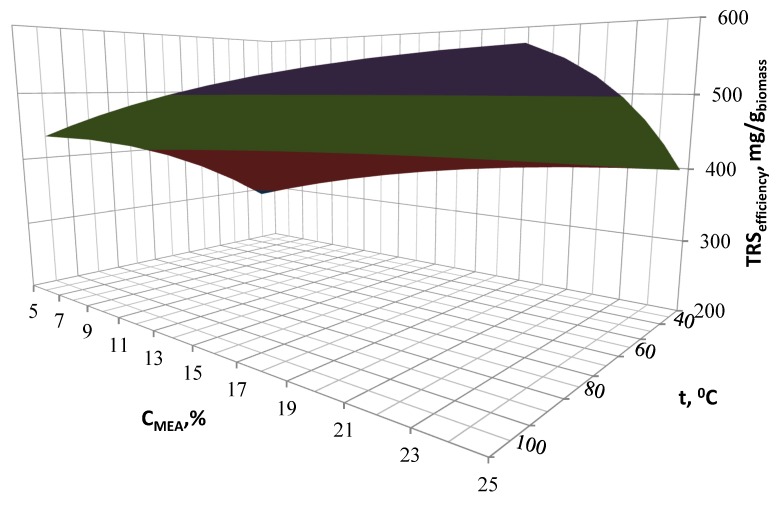
Influence of the pre-treatment temperature and MEA concentration on the TRS efficiency.

**Figure 6:**
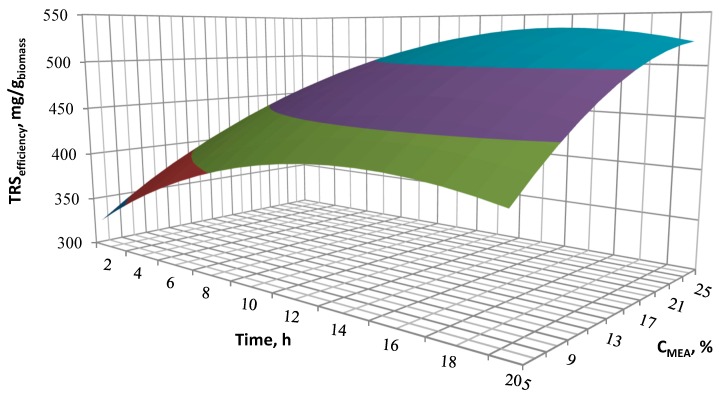
Influence of the pre-treatment time and MEA concentration on the TRS efficiency.

The effects of the designed process conditions were presented on the three-dimensional response surface area diagrams showing the influence and correlation of two variable input parameters on the TRS performance, with one variable being kept constant at the optimal level. Interactions of MEA concentration, reaction temperature and pre-treatment time for TRS efficiency are shown in Figure 4, Figure 5 and Figure 6.

Figure 4 presents the effect of temperature and time at a constant concentration of catalyst concentration. The yield of reducing sugars depends on the process temperature. The process temperature increase from 40 to 90 °C allows to obtain up to 1.5 times higher concentration of reducing sugars. A further increase in temperature no longer affects the TRS performance. The optimal process time is 14 h, extending the process time results in a decrease in efficiency, however the time influence is lower than the temperature influence. Extending the time from 2 h to 13 h increases the efficiency only by 1.2 times. 

Figure 5 shows the effect of concentration and time at a constant process temperature. The efficiency of TRS increases with an increase of MEA concentration. RSM analysis allowed to determine the optimal conditions for the alkaline pre-treatment. The effect of catalyst concentration and temperature at a constant time of 14 h is shown in Figure 6. The analysis of the surface of the response area confirms the magnitude of the effect of both concentration and temperature observed in Figure 4 and Figure 5. Both the increase in temperature and catalyst concentration have an influence on the increased efficiency of the cleavage of ester bonds linking lignin and hemicellulose. This has an effect on the biomass structure and thus the enhanced access of enzymes to the digestion of cellulose. It is determined that the optimal parameters for two-step hydrolysis are as follows: MEA concentration 21%, temperature 90 °C and reaction time of 14 h. A total content of reducing sugars of 528.7 mg/g_biomass_, including 462 mg/g_biomass_ of glucose are obtained for the optimal hydrolysate *H_opt_*. This result indicates that 85.7% of sugar polymers were hydrolyzed to simple sugars. However, it can be seen that the use of significantly milder processing conditions can lead to satisfactory performance results in sugars. This can be important in practical use, especially if process costs reductions are required. 

Comparing to other investigations [29] on the optimization of alkaline pre-treatment of poplar with NaOH as a catalyst, the degree of saccharification of biomass was achieved at 41.5% for NaOH concentration 2.8%, temperature of 94 °C and the time of 90 min [14]. 

### 3.5. Re-Use of MEA 

It is crucial to test the possibility of MEA re-use after the alkaline pre-treatment, as low-content waste process streams should be designed. Obtained results regarding the content of sugars in hydrolysates after the MEA reuse (Table 6) are presented in two groups, presenting the direct approach (according to Figure 1a) and the approach with a purification stage (according to Figure 1b). 

The compared processes were carried out under specified, repeatable and optimized conditions. The pre-treatment of biomass with an unpurified catalyst results in a significant reduction in the yield of TRS. The performance reduction can be observed particularly when glucose content obtained during P1, P2 and P3 are compared. The efficiency of glucose decreases almost twice in the second cycle of MEA re-usage. However, the subsequent use of the catalyst does not result in poorer performance of glucose in P2 and P3 cycles and does not seem to affect the yields of xylose, galactose, mannose, arabinose and cellobiose.

In the approach where the activated charcoal was used as a puryfing agent for the catalyst after P1O and P2O cycles, the similar TRS performance in subsequent treatment processes from subsequent biomass portions were obrained. Activated charcoal allows to sufficiently remove the absorbed chemical compounds in the catalyst solution, which allows to preserve its effectiveness for the biomass processing. In the purifying approach, the efficiency of sugars obtained in P1O, P2O and P3O cycles slightly drops. It can be concluded that MEA re-usage for hydrolysis is reasonable when the proposed purifying stage is applied.

Despite the possibility of re-using the catalyst solution, its loss is observed. The amount of the catalyst decreases by about 20–22% between cycles P1-P2 and P2-P3 in the direct approach, and by 25–30% between cycles P1O-P2O and P2O-P3O in the purifying approach. The loss is caused by the impossibility of complete removal of the catalyst from the pores of both biomass and activated carbon. Therefore, the loss is greater in the process with the purification stage (see Table 7).

### 3.6. Re-Use of Immobilized Enzymes

Immobilization allows a quick separation of enzymes from hydrolysates and enzymes recovery. However, the immobilization of cellulolytic enzymes on diatomaceous earth and their application on lignocellulosic materials is not widely described in the literature [45,78,79,80,81,82,83]. 

The compared processes were carried under specified, repeatable and optimized conditions. The pre-treatment of biomass with a re-used enzymatic catalyst results in a significant reduction in the yield of glucose, i.e., by 117.43 mg/g_biomass_ from E1 to E2 and to 333.38 from E2 to E3. However, the concentrations of other sugars increase, when re-used enzymes are applied during E2, and drops to 0.00 in E3. The effectiveness of biomass processing decreases to zero when the enzymes are to be re-used for the third time (see Table 8). These findings are a prelude to further work aimed at the material processing and enzymatic hydrolysis in a semi-continuous or continuous manner, the enzyme recovery may be further examined and developed, as the application of enzymes causes major costs incurred in the pre-treatment, saccharification and fermentation processes.

### 3.7. Dark Fermentation

Hydrogen production experiments are based on the mesophilic dark fermentation technique carried out periodically under anaerobic conditions. Hydrogen production is a very sensitive process, because it strongly depends on many factors, including, among others, the composition of the fermentation broth, substrate concentration, pH value and temperature [84]. 

**Figure 7:**
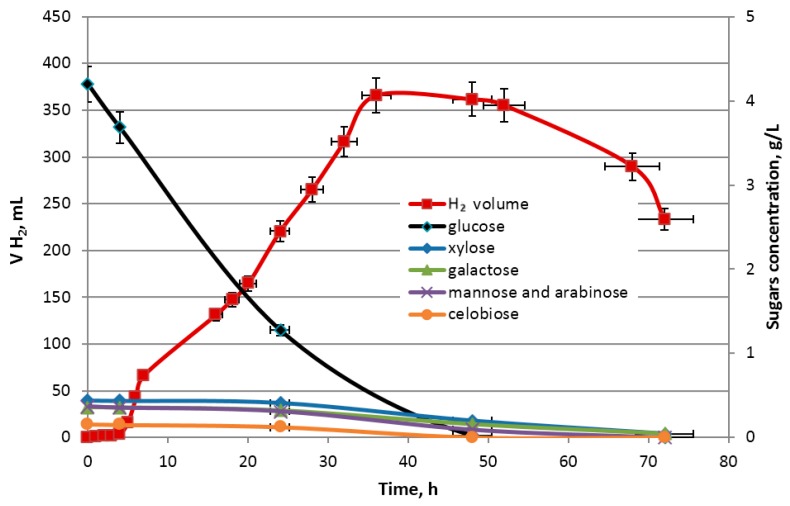
Changes in the volume of obtained hydrogen corresponding to changes of sugars concentrations during dark fermentation.

**Figure 8:**
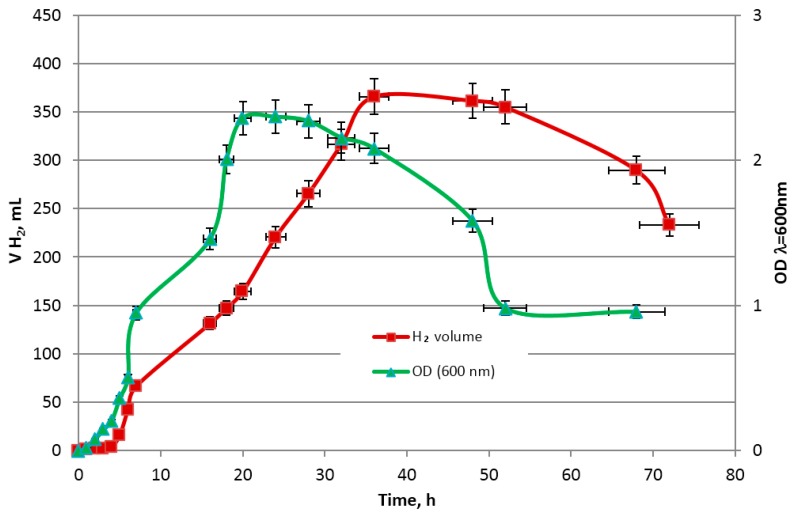
Changes in the volume of obtained hydrogen corresponding to changes OD_λ=600_ values during dark fermentation.

**Figure 9:**
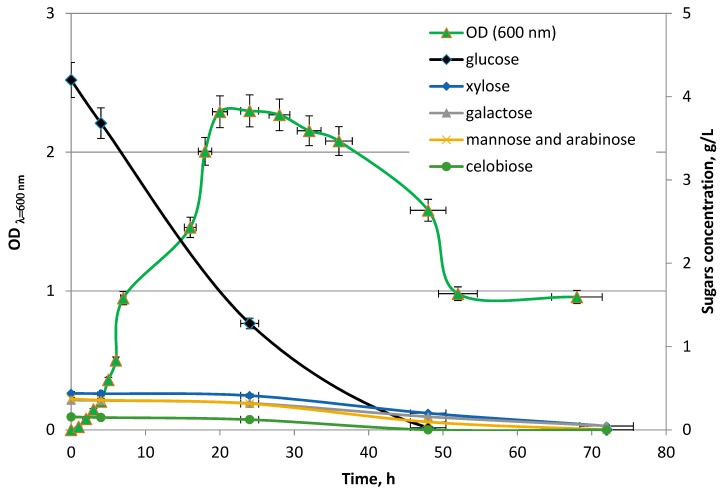
Changes in the sugar concentration corresponding to changes OD_λ=600_ values during dark fermentation.

The relation between the change in glucose, xylose, galactose, arabinose and mannose removal, optical density OD_λ = 600 nm_ as well as the evolution of the hydrogen concentration was investigated. The changes in the volume of obtained hydrogen gas corresponding to sugar concentration during the dark fermentation and the growth of *Enterobacter aerogenes* ATCC 13048 are presented in Figure 7 and Figure 8, respectively. The hydrogen production is positively correlated with the growth of *E. aerogenes* ATCC 13048 culture (Figure 8) and negatively correlated with the glucose concentration in the growth medium (Figure 7), which is the preferred source of carbon for biomass growth of this bacterial strain during the cultivation in the bioreactor. The decrease in xylose, mannose, arabinose, galactose and cellobiose concentrations were observed at the end of the logarithmic phase (Figure 9). A decrease in the cellobiose and other monosugar concentrations in the culture medium is observed, when glucose is depleted and catabolic repression ceases [85,86]. 

In addition to reducing sugars derived from the enzymatic hydrolysis of EP, the fermentation broth also contained potential fermentation inhibitors. However, analysis of the hydrolysates composition did not confirm the presence of the fermentation inhibitors, including HMF, levulinic acid, furfural or phenol derivatives constituting lignin degradation products. On the other hand, during the dark fermentation, apart from gaseous products, organic compounds were also formed, including acetic, butyric and succinic acids as well as ethanol (Table 9). During the dark fermentation, a higher concentration of acetic acid in relation to butyric acid is preferred for higher hydrogen yield [8,87,88]. In the logarithmic phase of growth, the concentration of formic acid increased from the initial value and then decreased in the stationary phase. It is possible in the presence of hydrogen decomposition of the formic acid occurred by formate hydrogen lyase [89,90]. By-products identified in the fermentation broths are presented in Table 9. 

The average amount of hydrogen generated during the dark fermentation carried by *Enterobacter aerogenes* ATCC 13048 in described processes was equal to 22.99 mL H_2_/g EP (0.6 mol H_2_/mol TRS). The efficiency of H_2_ production during the dark fermentation in this study seems to be quite low (0.6 mol H_2_/mol TRS), especially in comparison to the efficiency of H_2_ production in the model experiments of dark fermentation when glucose is used as a sole source of carbon [75,88,91,92,93,94,95]. However, this result should be compared with the analogous results obtained in real systems when the complex medium is used for growth of microorganism and hydrogen production. For example, a mixture of slaughterhouse waste and food industry residues allowed to obtain 16.5;mL H_2_/g waste in CSTR reactor inoculated with mixed cultures [96,97]. Diary manure fermentation with *Clostridium* sp. allowed to produce 31.5 mL/g waste [98]. It can be concluded that hydrogen yield production obtained during this study i.e., 22.99 mL H_2_/g EP is at the average level in comparison to analogous data reported in literature. 

## 4. Conclusions

The object of this study was to explore the possibility of reusing both types of catalysts during energy willow hydrolysis. The authors found that in order for the technology to be profitable it was necessary to undertake attempts to return catalysts. During the experiments it turned out that the preferred directions of research may vary depending on the approach, mainly due to the very high potential of the obtained hydrolysates. In order to use the raw material efficiently, it is necessary to analyze the potential economics as well as energetic effects of the pre-treatment process. Consequently, for each biofuel production, optimization should be carried out, preferably in a two-step manner.

Biomass must undergo the saccharification process prior to its fermentation. The need of lignocellulosic biomass pre-treatment results primarily from its composition. The efficiency of the saccharification process depends on the degree of lignin removal obtained during the biomass pre-treatment. It was found that higher glucose concentration in the hydrolysates corresponds with a higher degree of lignin removal. Therefore, the pre-treatment has a great improvement potential when monosugars efficiency is considered. Both the increase in temperature and the catalyst concentration have an influence on the better efficiency of the cleavage of ester bonds linking lignin and hemicellulose. This affects the biomass structure and thus allows for a better access of enzymes to the digestion of cellulose. It is determined that the optimal parameters for twostep hydrolysis are as follows: MEA concentration 21%, temperature 90 °C and reaction time of 14 h. A total content of reducing sugars obtained in optimal hydrolysate is 528.7 mg/g_biomass_. During the first step of pre-treatment, a wide matrix of value-added products may be obtained with high purity, as they are most commonly formed as a result of the transformation of lignin and hemicellulose derivatives. The changes occurring at this stage can and should definitely be an object of further research to make the process economically viable. 

It is concluded that MEA reuse is possible, while the possibility of enzyme recovery must be further examined and developed, as it is a major part of the costs incurred in the multi-step biomass processing. As the approach concerning enzymatic hydrolysis increases the hydrolysis cost, a two–stage pre-treatment procedure is not economically profitable. On this premise, it is of no practical significance to study the optimization of technological conditions without taking into account the possibility of acquisition and purification of lignin derivatives. The enzymatic hydrolysis should be carried in a possibly simple matrix—after lignin and hemicelulose derivatives separation, as the presence of some chemical compounds may cause an inhibitory effect on the enzymes. Finally, the specific products, namely saccharides of cellulose and hemicellulose must be easily separated from other hydrolysis products in order to be used in bioconversion processes, as the presence of HMF or levulinic acid may inhibit the dark fermentation process. 

The average amount of hydrogen generated during fermentation carried by *Enterobacter aerogenes* ATCC 13048 in this study was equal to 0.6 mol H_2_/mol TRS (22.99 mL H_2_/g EP). The proposed approach of optimization of alkaline delignification, enzymatic saccharification, followed by dark fermentation allows to produce satisfying yields of hydrogen in a laboratory scale. The resulting amount of hydrogen is similar to the average results obtained in comparable studies described in the literature for hydrogen production by means of dark fermentation with the use of such complex media such as diary manure, rice winery wastewater, organic waste containing sucrose or xylose [92,98,99,100,101,102,103].

## Figures and Tables

**Table 1:** Composition of selected lignocellulosic feedstock materials [10,22].

Material	% Glucan	% Xylan	% Arabinan	% Lignin	% Ash	% Extractives
Empty palm fruit bunch	38.00	11.00	0.13	40.00	9.37	1.50
Rice husk	41.00	12.00	0.50	25.00	16.00	5.50
Pine tree wood	40.00	20.50	1.60	36.60	1.25	0.05
Energy poplar	47.00	18.00	0.90	29.00	1.40	3.70

**Table 2:** Hydrogen yield obtained during fermentation of lignocellulosic hydrolysates after different pre-treatment approaches.

Type of Lignocellulosic Biomass	Pretreatment Method	Inoculum During Dark Fermentation	T (°C)	H_2_ Yield (mmol H_2_/g of Substrate)	Reference
Corn stalk	Lime loading of 0.10 g/g of biomass for 96 h	microflora from rotted wood	60	5.69	[42]
Corn stover	Microwave assisted acid pretreatment (H_2_SO_4_ 0.3 N for 45 min)	anaerobic sludge	55	0.68	[43]
Cornstalk	NaOH at 120 °C for 20 min	anaerobic sludge	55	1.70	[44]
Rice straw	10% ammonia and 1.0% H_2_SO_4_	*T. neapolitana*	75	2.70	[13]
Wheat straw	HCl pretreated	cow dung/compost	36	3.04	[45]
Cornstalk	H_2_SO_4_ 0.5% at 121 °C for 60 min	microwave irradiated cow dung compost	36	6.44	[46]
Grass	4% HCl	anaerobic	35	2.86	[15]

**Table 3:** Input variables for the Box-Behnken design during MEA pre-treatment.

Variable	Unit	Symbol	Coding Level		
			−1	0	1
MEA concentration	% (*v*/*v*)	X_1_	5	15	25
Process temperature	°C	X_2_	40	70	100
Reaction time	h	X_3_	2	11	20

**Table 4:** Changes in the biomass content [%] occurring during alkaline pre-treatment.

Exp No.	Cellulose Content	Hemicellulose Content	Lignin Content	Biomass Recover	Lignin Removal	Glucan Recovery	Hemicellulose Recovery
1	42.3	15.3	24.4	84.7	24.4	90.6	71.4
2	42.8	13.9	23.7	81.6	29.4	88.4	62.9
3	43.1	15.4	22.8	77.1	35.8	84.1	65.5
4	51.3	12.4	15.7	69.5	60.2	90.2	47.8
5	41.8	15.7	25.5	85.0	20.7	90.1	73.6
6	39.7	13.6	22.2	76.7	37.9	77.2	57.8
7	45.4	15.8	24.9	80.3	26.9	92.4	70.2
8	49.7	12.9	22.2	76.5	37.9	96.2	54.5
9	38.4	12.7	24.3	88.1	21.9	85.5	61.7
10	45.1	13.9	21.7	76.5	39.3	87.4	58.5
11	39.4	13.0	22.4	81.5	33.3	81.3	58.7
12	47.6	12.9	21.1	72.9	43.8	87.8	52.0
13	46.0	14.6	21.1	77.5	40.3	90.2	62.3
14	47.6	14.9	21.1	75.0	42.3	90.3	61.8
15	48.0	15.0	21.1	76.5	41.0	92.9	63.3

**Table 5:** Box-Behnken experimental design for two-step hydrolysis of EP and enzymatic hydrolysis of raw EP.

Exp No.	MEA Concentration	t	Time	Glucose Concentration	Total Sugar Concentration
	% [*v*/*v*]	[°C]	[h]	[mg/g_biomass_]	[mg/g_biomass_]
**1**	5	40	11	232.91	298.21
**2**	25	40	11	327.97	415.42
**3**	5	100	11	364.99	426.94
**4**	25	100	11	471.77	515.46
**5**	5	70	2	236.08	309.77
**6**	25	70	2	327.97	407.84
**7**	5	70	20	287.63	357.62
**8**	25	70	20	426.21	501.81
**9**	15	40	2	234.49	307.38
**10**	15	100	2	342.23	418.82
**11**	15	40	20	263.01	333.71
**12**	15	100	20	451.56	504.00
**13**	15	70	11	388.18	465.84
**14**	15	70	11	381.00	462.61
**15**	15	70	11	372.34	455.04
**Raw**	15	100	20	147.25	189.22

**Table 6:** Statistical parameters for the model coefficients based on the Box-Behnken design.

Coefficient	Estimated Value	Standard Deviation	*T*-Value	*p*-Value
**β0**	−25.654	48.205	−0.532	0.611
**β1**	12.322	2.281	5.402	0.001
**β2**	5.522	1.201	4.597	0.002
**β3**	12.074	2.790	4.328	0.003
**β11**	−0.224	0.074	−3.019	0.019
**β22**	−0.029	0.008	−3.464	0.010
**β33**	−0.562	0.092	−6.138	0.000
**β23**	0.054	0.026	2.056	0.079

**Table 7:** Content of monosugars in hydrolysates after MEA re-use.

No.	Glucose Content	Xylose Content	Galactose Content	Mannose, Arabinose Content	Cellobiose Content	Catalyst amount Reduction [%]
	[mg/g_biomass_]					
Direct approach						
P1	462.6	33.6	18.9	13.7	6.0	20
P2	240.1	30.2	19.0	11.6	7.5	22
P3	238.9	25.6	15.4	10.1	8.1	22
Purification approach						
P1O	456.4	34.1	18.3	14.0	4.5	25
P2O	440.0	33.4	18.0	13.5	5.0	28
P3O	423.6	31.8	17.8	13.3	5.2	30

Where P1/2/3—1st/2nd/3rd cycle of MEA usage in direct approach, P1/2/3O—1st/2nd/3rd cycle of MEA usage in purifying approach.

**Table 8:** Content of monosugars in hydrolysates after the enzyme’s recovery.

No.	Glucose Content	Xylose Content	Galactose Content	Mannose, Arabinose Content	Cellobiose Content	Catalyst Amount Reduction [%]
	[mg/g_biomass_]					
E1	450.81	23.04	14.42	10.66	5.11	35
E2	333.38	27.36	32.93	47.88	6.08	33
E3	0.00	0.00	0.00	0.00	0.00	36

Where: E1/2/3—1st/2nd/3rd cycle of immobilized enzymes usage.

**Table 9:** Formation of by-products during fermentation.

Sample	Succinic Acid [g/L]	Formic Acid [g/L]	Acetic Acid [g/L]	Butyric Acid [g/L]	Ethanol [g/L]
Initial	0.00	0.21	0.17	0.00	0.00
Final	0.46	0.12	1.55	0.21	1.12
